# Preoperative identification of microvascular invasion in hepatocellular carcinoma by XGBoost and deep learning

**DOI:** 10.1007/s00432-020-03366-9

**Published:** 2020-08-27

**Authors:** Yi-Quan Jiang, Su-E Cao, Shilei Cao, Jian-Ning Chen, Guo-Ying Wang, Wen-Qi Shi, Yi-Nan Deng, Na Cheng, Kai Ma, Kai-Ning Zeng, Xi-Jing Yan, Hao-Zhen Yang, Wen-Jing Huan, Wei-Min Tang, Yefeng Zheng, Chun-Kui Shao, Jin Wang, Yang Yang, Gui-Hua Chen

**Affiliations:** 1 Department of Hepatic Surgery and Liver Transplantation Center, The Third Affiliated Hospital, Organ Transplantation Institute, Sun Yat-Sen University, 600 Tianhe Road, Guangzhou, 510630 Guangdong China; 2 Department of Radiology, The Third Affiliated Hospital, Sun Yat-Sen University, 600 Tianhe Road, Guangzhou, 510630 China; 3 Tencent Youtu Lab, Malata Building, Kejizhongyi Road, Nanshan District, Shenzhen, 518075 China; 4 Department of Pathology, The Third Affiliated Hospital, Sun Yat-Sen University, 600 Tianhe Road, Guangzhou, 510630 China; 5 Tencent Healthcare, Tengxun Building, Kejizhongyi Road, Nanshan District, Shenzhen, 518075 China; 6 Organ Transplantation Research Center of Guangdong Province, Guangzhou, 510630 Guangdong China; 7 Guangdong Key Laboratory of Liver Disease Research, The Third Affiliated Hospital, Sun Yat-Sen University, 600 Tianhe Road, Guangzhou, 510630 Guangdong China

**Keywords:** Hepatocellular carcinoma, Micro-vascular invasion, Deep learning, Neural network models, Radiomics

## Abstract

**Purpose:**

Microvascular invasion (MVI) is a valuable predictor of survival in hepatocellular carcinoma (HCC) patients. This study developed predictive models using eXtreme Gradient Boosting (XGBoost) and deep learning based on CT images to predict MVI preoperatively.

**Methods:**

In total, 405 patients were included. A total of 7302 radiomic features and 17 radiological features were extracted by a radiomics feature extraction package and radiologists, respectively. We developed a XGBoost model based on radiomics features, radiological features and clinical variables and a three-dimensional convolutional neural network (3D-CNN) to predict MVI status. Next, we compared the efficacy of the two models.

**Results:**

Of the 405 patients, 220 (54.3%) were MVI positive, and 185 (45.7%) were MVI negative. The areas under the receiver operating characteristic curves (AUROCs) of the Radiomics-Radiological-Clinical (RRC) Model and 3D-CNN Model in the training set were 0.952 (95% confidence interval (CI) 0.923–0.973) and 0.980 (95% CI 0.959–0.993), respectively (*p* = 0.14). The AUROCs of the RRC Model and 3D-CNN Model in the validation set were 0.887 (95% CI 0.797–0.947) and 0.906 (95% CI 0.821–0.960), respectively (*p* = 0.83). Based on the MVI status predicted by the RRC and 3D-CNN Models, the mean recurrence-free survival (RFS) was significantly better in the predicted MVI-negative group than that in the predicted MVI-positive group (RRC Model: 69.95 vs. 24.80 months, *p* < 0.001; 3D-CNN Model: 64.06 vs. 31.05 months, *p* = 0.027).

**Conclusion:**

The RRC Model and 3D-CNN models showed considerable efficacy in identifying MVI preoperatively. These machine learning models may facilitate decision-making in HCC treatment but requires further validation.

**Electronic supplementary material:**

The online version of this article (10.1007/s00432-020-03366-9) contains supplementary material, which is available to authorized users.

## Introduction

Liver cancer is the sixth-most common cancer in the world and the fourth cause of cancer-related death worldwide (Villanueva 2019). Throughout the world, ~ 841,000 people are diagnosed with hepatocellular carcinoma (HCC), and ~ 782,000 people die from HCC each year (Bray et al. 2018). The mainstay treatment for HCC is surgery, including hepatic resection and liver transplantation. Despite receiving radical surgery, patients still have a high risk of recurrence; thus, an accurate preoperative cancer assessment are essential for determining the appropriate surgical approach and management strategy to decrease the recurrence rate.

Recent studies have proposed the importance of a preoperative assessment of microvascular invasion (MVI), which can be used to guide therapy in patients with HCC (Banerjee et al. 2015; Cucchetti et al. 2010; Hyun et al. 2018; Lee et al. 2017; Renzulli et al. 2016; Wang et al. 2018a; Wu et al. 2015; Xu et al. 2019). Studies have shown that MVI is an independent histopathological prognostic factor associated with survival in all-stage HCC patients (Mazzaferro et al. 2009). Furthermore, MVI has been reported to be a better predictor of tumour recurrence and overall survival than the Milan criteria (Lim et al. 2011). For patients with MVI, a more aggressive treatment strategy may be preferred, such as a wide resection margin or anatomical resection for patients receiving hepatic resection (HR), an ablation margin of at least 0.5–1 cm 360° around the tumour for patients receiving ablation, and neoadjuvant therapy before surgery (Hirokawa et al. 2014; Hocquelet et al. 2016; Nakazawa et al. 2007; Nault et al. 2018; Zhao et al. 2017). For liver transplantation (LT) in patients with HCC, MVI status has been recognized as an essential variable for identifying patients who will benefit most from LT (Mazzaferro et al. 2009, 2018).

However, the traditional method of identifying MVI is based on postoperative microscopic examination of surgical specimens even though the most important treatment decisions are commonly determined before surgery. Therefore, exploring new methods that can be used to preoperatively assess MVI to determine the most appropriate treatment strategy for HCC patients is important. Developments in imaging technology have enabled non-invasive assessments of MVI preoperatively (Banerjee et al. 2015; Hyun et al. 2018; Lee et al. 2017; Renzulli et al. 2016; Wang et al. 2018a; Wu et al. 2015; Xu et al. 2019; Zheng et al. 2017).

Advances in imaging technology, together with artificial intelligence (Bi et al. 2019), have allowed researchers to create various diagnostic and treatment models and improved the diagnostic efficacy in liver cancer, dermatology, ophthalmology, lung and breast cancers, neurology, cardiovascular diseases, gastrointestinal endoscopy, and genetic diseases, etc. (Attia et al. 2019; Chilamkurthy et al. 2018; Coudray et al. 2018; Esteva et al. 2017; Gurovich et al. 2019; Kermany et al. 2018; Mori et al. 2018; Rampasek and Goldenberg 2018; Yasaka et al. 2018; Zou et al. 2019). The purpose of the current study is to develop models using eXtreme Gradient Boosting (XGBoost) and deep learning to provide a preoperative non-invasive assessment method for MVI in HCC patients. An artificial intelligence system for hepatology requires a great amount of work, but it is just the beginning of the dramatic change that artificial intelligence will bring about in medicine.

## Materials and methods

This retrospective clinical study was approved by our institutional review board. Because of the retrospective nature of the study, patient consent for inclusion was waived. All private information of the included patients was erased.

### Case cohort

A retrospective cohort from collected from 2010 to 2018 was analysed. The inclusion and exclusion criteria were as follows: (1) histological diagnosis of HCC; (2) HR or LT received as primary therapy; (3) preoperative four-phase contrast-enhanced computed tomography (CT) performed 2 months at most before LT or HR; and (4) available postoperative pathologic specimens. Details about pathological assessment of MVI and CT imaging protocol are shown in Supplemental methods.

### Methods overview

The traditional method of assessing MVI status preoperatively is to manually collect radiological features, radiomics features and clinical variables and develop a predictive model based on such collected information. Such models are more interpretable but require more manpower and materials. Nowadays, deep learning models excel at automated image recognition with high efficiency and accuracy. In the current study, we developed predictive models by XGBoost in the traditional way and also developed a predictive model based on an emerging algorithm, namely, deep learning, and compared the efficacy of the two methods.

### Predictive models based on XGBoost (Chen and Guestrin 2016)

#### Tumor segmentation

Tumor segmentation was manually and independently performed by three radiologists (A, B and C) (all of the radiologists had at least 3 years of experience in HCC diagnosis) for the three phases of the volume data (the AP, PVP, and DP), and the results were reviewed by a radiologist (D) with 20 years of experience in HCC diagnosis. The segmentation boundaries were drawn with ITK-SNAP software (https://www.radiantviewer.com) slice-by-slice for each volume along the visible borders of the lesion. The 3D segmentation of the tumor provides the volume-of-interest (VOI) for the later feature extraction step.

#### Radiomics feature extraction

Radiomics is defined as the quantitative mapping, that is, the extraction, analysis and modelling of many medical image features in relation to prediction targets. The fundamental principle of radiomics is to extract high-dimension features, e.g., first-, second-, and higher-order statistics, to quantitatively describe the attributes of the VOI based on tomographic data. In the current study, the VOI was the 3D tumor region that was manually segmented from the CT scan. The radiomics features were extracted from the tumor VOI (VOI-full) and 1 cm extended from the VOI boundary (VOI-extension) via standard morphology binary dilation. To guarantee the extension of the tumor boundary inside the liver region, we obtained the liver mask from an automatic liver organ segmentation algorithm and discarded the non-liver regions outside the mask. The segmentation of a typical case is shown in Fig. 2. We used the open source PyRadiomics package for radiomics feature extraction. For each volume of the 3 different phases, we extracted 1217 features from the VOI-full and VOI-extension regions, consisting of a set of 7302 radiomics features.

#### Radiological feature extraction

The radiological features of the four-phase CT images of all cases were extracted and summarized by the aforementioned radiologists, and during this process, they were blinded to the pathological and clinical data. Next, the controversial cases among the three radiologists (A, B, C) were jointly evaluated until a final consensus was reached, and then they were finally reviewed by the most senior radiologist (D).

The extracted radiological features are a semantic interpretation of the tumor regions by the radiologists organized in a binary format. The summary of the radiological features were as follows: (1) liver morphology (normal vs. cirrhosis); (2) number of hepatic lobes involved (one lobe involved vs. two or more lobes involved); (3) number of tumors (one vs. more than one); (4) peritumoral satellite nodule (absence vs. presence); (5) maximum diameter of tumor (> 5 cm vs. ≤ 5 cm); (6) tumor growth pattern (intrahepatic growth vs. extrahepatic growth); (7) intratumoral hemorrhage (absence vs. presence); (8) intratumoral necrosis (absence vs. presence); (9) tumor margin (smooth vs. nonsmooth); (10) enhancing “capsule” (absence vs. presence); (11) AP hyperenhancement (absence vs. presence); (12) internal arteries (absence vs. presence); (13) peritumoral enhancement (absence vs. presence); (14) mosaic pattern or nodule-in-nodule pattern (absence vs. presence); (15) nonperipheral washout (absence vs. presence); (16) hypodense halos (absence vs. presence); and (17) tumor steatosis (absence vs. presence). The largest nodule was evaluated if multiple nodules existed. The definitions of some radiological features are shown in Supplemental methods.

#### Clinical variables

Baseline data of the patients including age, sex, background liver disease, diabetes, surgery type, primary tumor size, tumor count, *α*-fetoprotein (AFP) level, aspartate aminotransferase (AST) level, alanine aminotransferase (ALT) level, prothrombin time (PT), international normalized ratio (INR), serum fibrinogen (FBG) level, platelet (PLT) count, total bilirubin (TBIL) level, serum creatinine (Scr) level, Child–Pugh class, MELD score were recorded.

#### Feature analysis and predictive model based on XGBoost

Using XGBoost, we developed MVI prediction models based on radiological features (the Radiological Model), radiomics features (Radiomics Model) and a combination of radiological features, radiomics features, and clinical variables (Radiological-Radiomics-Clinical (RRC) Model). Details about XGBoost model are shown in Supplemental methods.

### Deep learning: the 3D-CNN predictive model (Wang et al. 2018b)

**Fig. 1:**
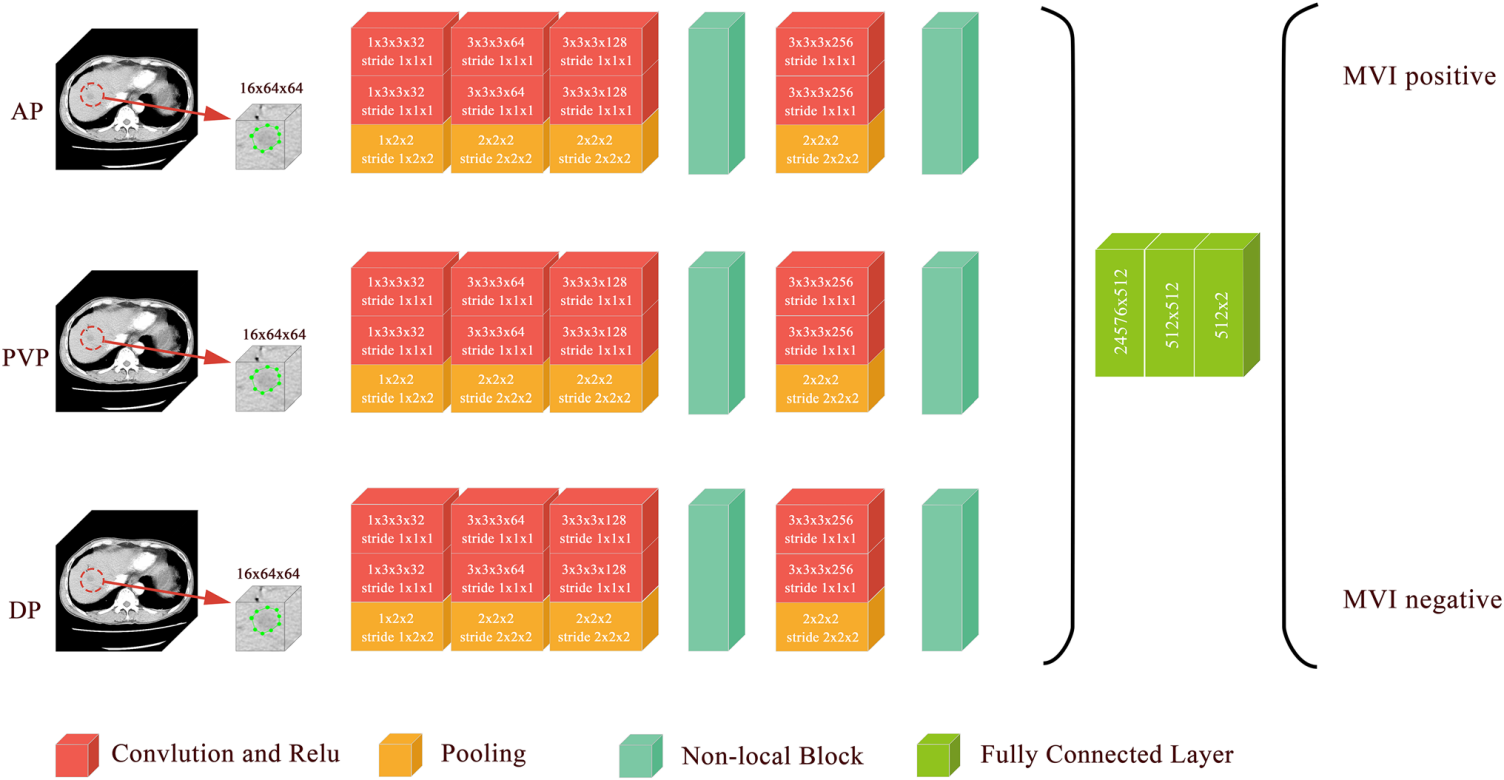
Schematic of the 3D-CNN model for the prediction of MVI status

Convolutional neural networks excel at medical image recognition (Hosny et al. 2018; Litjens et al. 2017). A 3D-CNN Model was developed to assess MVI in an end-to-end training fashion, in which feature extraction and predictive model construction were automatically processed by a single neural network. We developed several empirical principles to process the input data and guide the design of the deep neural networks: (1) the input should be a small volume sample that is mostly covered by the tumour region to exclude interference from nearby tissues; 2) the input should be sampled within the tumour region to force the network to learn the relevant features of the tumour; and (3) the depth of the CNN should not be profound to avoid the overfitting problem due to the limited size of the training cohort. According to these principles, we proposed a CNN as shown in Fig. 1. The network takes three 16 × 64 × 64 patches as input and passes them through several intermediate layers to extract deep features, which are further fused and fed into the decision layers to generate the final MVI assessment result. Details about CNN model are shown in Supplemental methods.

### Statistical analysis

The performance of the predictive models was evaluated by the areas under the receiver operating characteristic curve (AUROC) and precision recall curve (AUPRC). The accuracy, sensitivity, specificity, positive predictive value, negative predictive value and f1 score of the models were also calculated and are presented. Hanley and McNeil analysis was performed to compare the efficacy of the proposed models. Recurrence-free survival analyses were performed based on the MVI status predicted by the XGBoost and 3D-CNN models. Recurrence-free survival was defined as the time from the surgery to local, regional, or distant cancer relapse or to death due to HCC.

## Results

Of the 1618 patients with a diagnosis of HCC at the * between 2010 and 2018, a total of 405 patients met the inclusion criteria (flow chart is shown in Supplemental Fig. 1). Of the 405 patients, 220 patients (54.3%) were MVI positive, and 185 patients (45.7%) were MVI negative. The baseline characteristics of all patients are presented in Table 1. All patients were randomly assigned to the training set and validation set at a ratio of 8:2. The radiological features and baseline characteristics of patients stratified by MVI status are presented in Table 2 and Supplemental Table 1, respectively.

**Table 1:** Baseline information of the entire cohort

Variables	All patients (*n* = 405)
Age, years	48.5 ± 13.4
Sex (male)	344 (84.9%)
Diabetes (yes)	36 (8.9%)
Background liver disease	
HBV infection	346 (85.4%)
Other	59 (14.6%)
Surgery type	
HR	347 (85.7%)
LT	58 (14.3%)
BCLC stage	
0	13 (3.2%)
A	221 (54.6%)
B	104 (25.7%)
C	67 (16.5%)
AFP	
< 10	121 (29.9%)
10–100	78 (19.3%)
100–400	61 (15.1%)
400–1000	24 (5.9%)
> 1000	121 (29.9%)
MVI status	
Positive	220 (54.3%)
Negative	185 (45.7%)
ALT	54.1 ± 98.7
AST	62.7 ± 156.6
PLT	180.8 ± 85.5
PT	14.4 ± 2.2
INR	1.13 ± 0.24
FBG	3.3 ± 1.2
ALB	39.1 ± 5.0
TBIL	27.7 ± 90.9
SCr	62.3 ± 66.4
Child–Pugh class	
A	340 (84.0%)
B	54 (13.3%)
C	11 (2.7%)
MELD score	7.9 ± 4.3

*HBV* hepatitis B virus, *HR* hepatic resection, *LT* liver transplantation, *BCLC* Barcelona Clinic Liver Cancer, *AFP* *α*-fetoprotein, *ALT* alanine aminotransferase, *AST* aspartate aminotransferase, *PLT* platelet, *PT* prothrombin time, *INR* international normalized ratio, *FBG* fibrinogen, *ALB* albumin, *TBIL* total bilirubin, *SCr* serum creatinine, *MELD* model for end-stage liver disease

**Table 2:** Radiological features stratified by MVI status in the training set and validation set

Variable	Training set			Validation set			*p*^a^
	MVI negative (*n* = 148)	MVI positive (*n* = 176)	*p*	MVI negative (*n* = 37)	MVI positive (*n* = 44)	*p*	
Tumour count			0.003			0.06	0.46
1	103	95		27	21		
2	24	25		1	7		
3	4	10		3	3		
> 3	17	46		6	13		
Liver cirrhosis			0.67			0.56	0.84
No	53	59		12	17		
Yes	95	117		25	27		
Lobes involved			< 0.001			0.001	0.80
0	108	90		31	21		
1	40	86		6	23		
Tumour growth pattern			0.024			0.63	0.53
Intrahepatic growth	142	157		34	39		
Extrahepatic growth	6	19		3	5		
Satellite nodule			< 0.001			0.02	0.91
No	123	114		32	28		
Yes	25	62		5	16		
Intratumour haemorrhage			0.27			0.82	0.90
No	127	143		31	36		
Yes	21	33		6	8		
Intratumour necrosis			0.001			< 0.001	1.00
No	86	69		27	13		
Yes	62	107		10	31		
Margin of the tumour			< 0.001			< 0.001	0.60
Smooth	88	26		21	5		
Not smooth	60	150		16	39		
Pseudocapsule			0.19			0.08	0.53
Well-defined	53	51		17	12		
Ill-defined	95	125		20	32		
AP hyperenhancement			0.11			0.99	0.54
No	21	15		5	6		
Yes	127	161		32	38		
Internal arteries			< 0.001			< 0.001	1.00
No	96	47		27	10		
Yes	52	129		10	34		
Peritumoural enhancement			< 0.001			0.02	0.46
No	137	131		36	35		
Yes	11	45		1	9		
Mosaic pattern			< 0.001			0.08	0.35
No	60	36		13	8		
Yes	88	140		24	36		
Presence of wash out			0.36			0.99	0.65
No	20	18		5	6		
Yes	128	158		32	38		
Hypo-dense halo			0.003			0.11	0.04
No	130	170		29	40		
Yes	18	6		8	4		
Max diameter (mm)			< 0.001			< 0.001	0.73
> 50	49	114		6	33		
≤ 50	99	62		31	11		
Steatosis of the tumour			0.88			0.66	0.11
No	145	172		36	42		
Yes	3	4		1	2		

*p*^a^, *p* value for the test between the training set and the validation set

### Development of an MVI prediction model based on the 3D-CNN

**Fig. 2:**
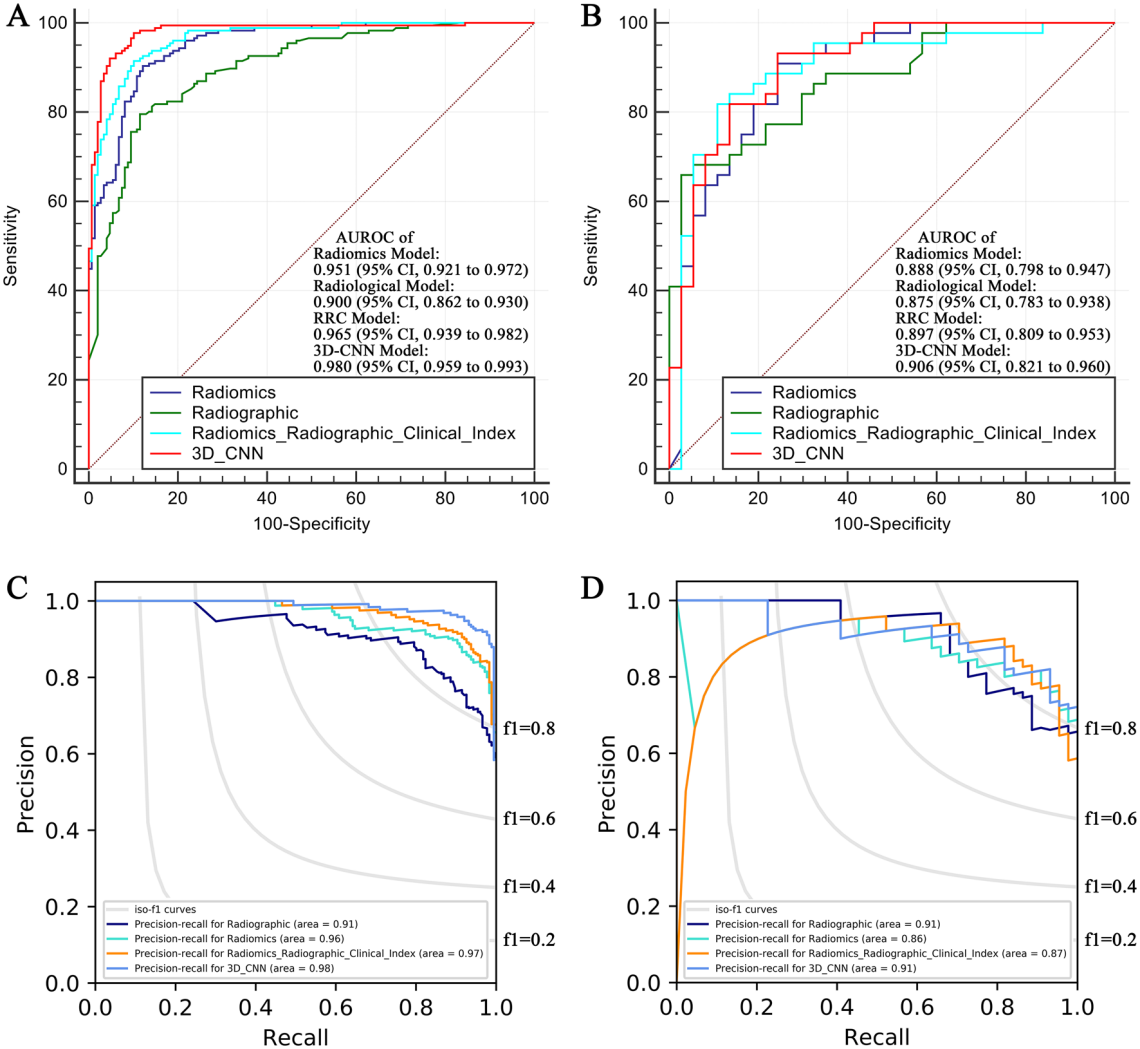
Performance of the predictive models. **a** The ROC curve of the predictive models in the training set. **b** The ROC curve of the predictive models in the validation set. **c** The PRC curve of the predictive models in the training set. **d** The PRC curve of the predictive models in the validation set

In the current study, a 3D-CNN Model was developed to assess MVI in an end-to-end training fashion. A graphical abstract of the 3D-CNN Model is shown in Supplemental Fig. 2, and the detailed schematic of the 3D-CNN Model developed to predict MVI status is shown in Fig. 1. The performance of the 3D-CNN Model for the identification of MVI is presented in Table 3. The AUROC values of the 3D-CNN Model in the training set and the validation set were 0.980 (95% CI 0.959–0.993) and 0.906 (95% CI 0.821–0.960), respectively (Fig. 2a, b). The AUPRC values of the 3D-CNN Model in the training set and the validation set were 0.99 and 0.90, respectively (Fig. 2c, d). To improve the interpretability of the 3D-CNN model, we attempted to predict the 15 most important variables selected by the XGBoost method and some valuable radiological features of HCC based on the 3D-CNN Model. A high prediction accuracy means that the established CNN model has encoded the interpretable characteristics to assist in the decision-making process in predicting MVI status. The performance of the 3D-CNN Model in predicting these features is presented in Supplemental Table 2. For example, the AUROC, specificity and sensitivity were 0.776, 0.923 and 0.564, respectively, in predicting the tumor margin status using the 3D-CNN Model.

**Table 3:** Performance of the MVI predictive models in the validation set

Model	AUROC (training set)	AUROC (validation set)	Specificity	Sensitivity	Accuracy	Positive predictive value	Negative predictive value	F1 score
Radiological Model	0.900	0.875	0.973	0.659	0.802	0.967	0.706	0.784
Radiomics Model	0.951	0.888	0.757	0.909	0.840	0.816	0.875	0.860
RRC Model	0.965	0.897	0.892	0.818	0.852	0.900	0.805	0.857
3D-CNN Model	0.980	0.906	0.757	0.932	0.852	0.820	0.903	0.872

### Development of MVI predictive models based on XGBoost (Chen and Guestrin 2016)

**Fig. 3:**
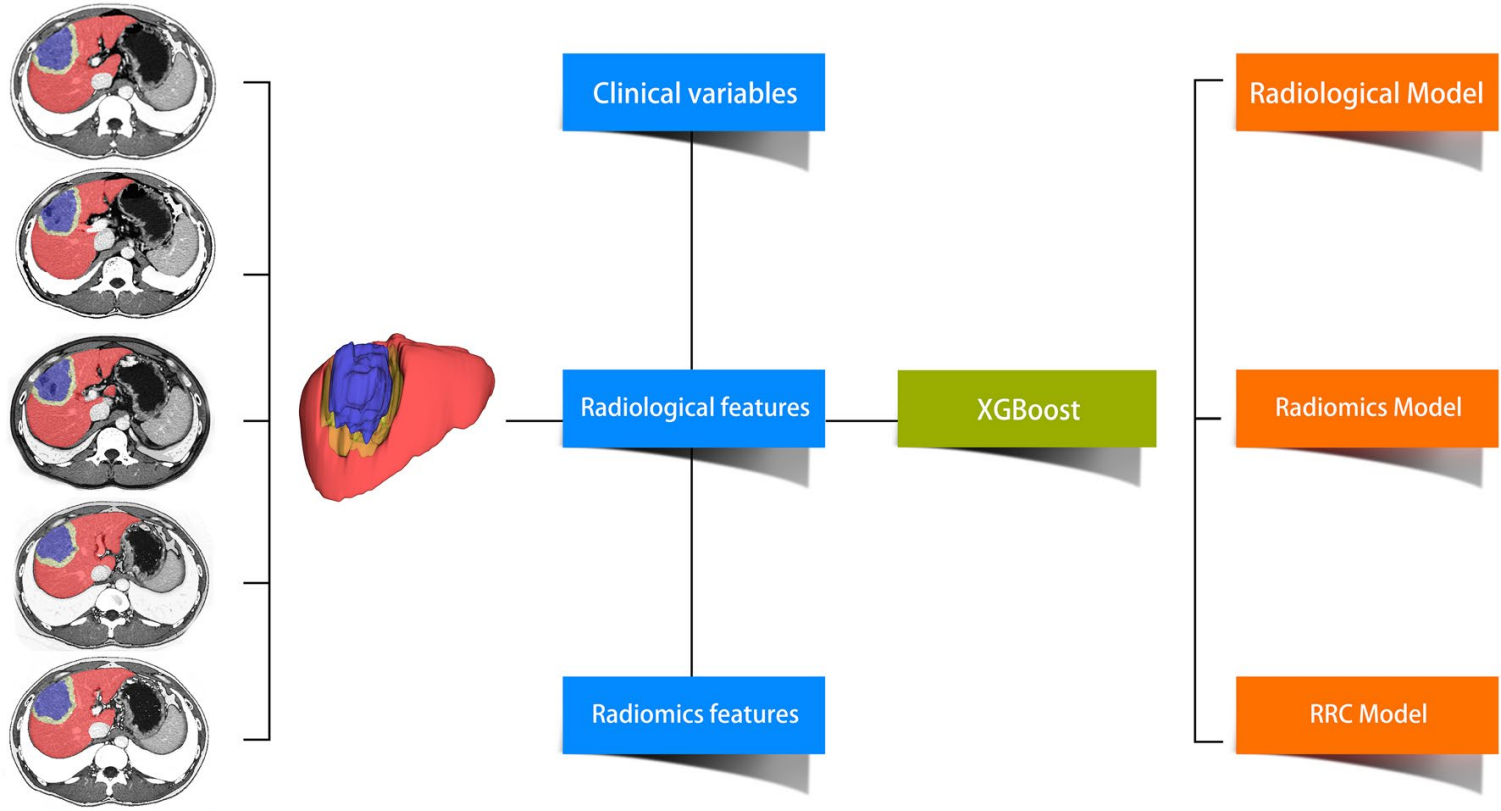
Schematic of the models developed by XGBoost. The liver was automatically segmented by an automatic segmentation algorithm (red part), and the non-liver part of the image was discarded. Then, tumor segmentation was completed for each slice by radiologists. The radiomics features were extracted from the tumor VOI (VOI-full, blue part) and 1 cm extended from the VOI boundary (VOI-ext, yellow part) via standard volume boundary erosion expansion

Next, we used traditional methods to access MVI status preoperatively, that is, manually collecting images and clinical information and developing predictive models based on such collected information. We developed MVI prediction models based on radiological features (Radiological Model), radiomics features (Radiomics Model), clinical variables and their combinations (Radiomics-Radiological-Clinical Model, RRC Model) (Fig. 3). The performance of the predictive models generated by XGBoost is also presented in Table 3. The areas under the receiver operating characteristic curves (AUROCs) of the Radiological Model in the training set and the validation set were 0.900 (95% CI 0.776–0.862) and 0.875 (95% CI 0.761–0.925), respectively. The AUROC values of the Radiomics Model in the training set and the validation set were 0.948 (95% CI 0.918–0.969) and 0.873 (95% CI 0.781–0.937), respectively. The AUROC values of the RRC Model in the training set and the validation set were 0.952 (95% CI 0.923–0.973) and 0.887 (95% CI 0.797–0.947), respectively (Fig. 2).

### Importance ranking of variables for predicting MVI status by XGBoost

**Fig. 4:**
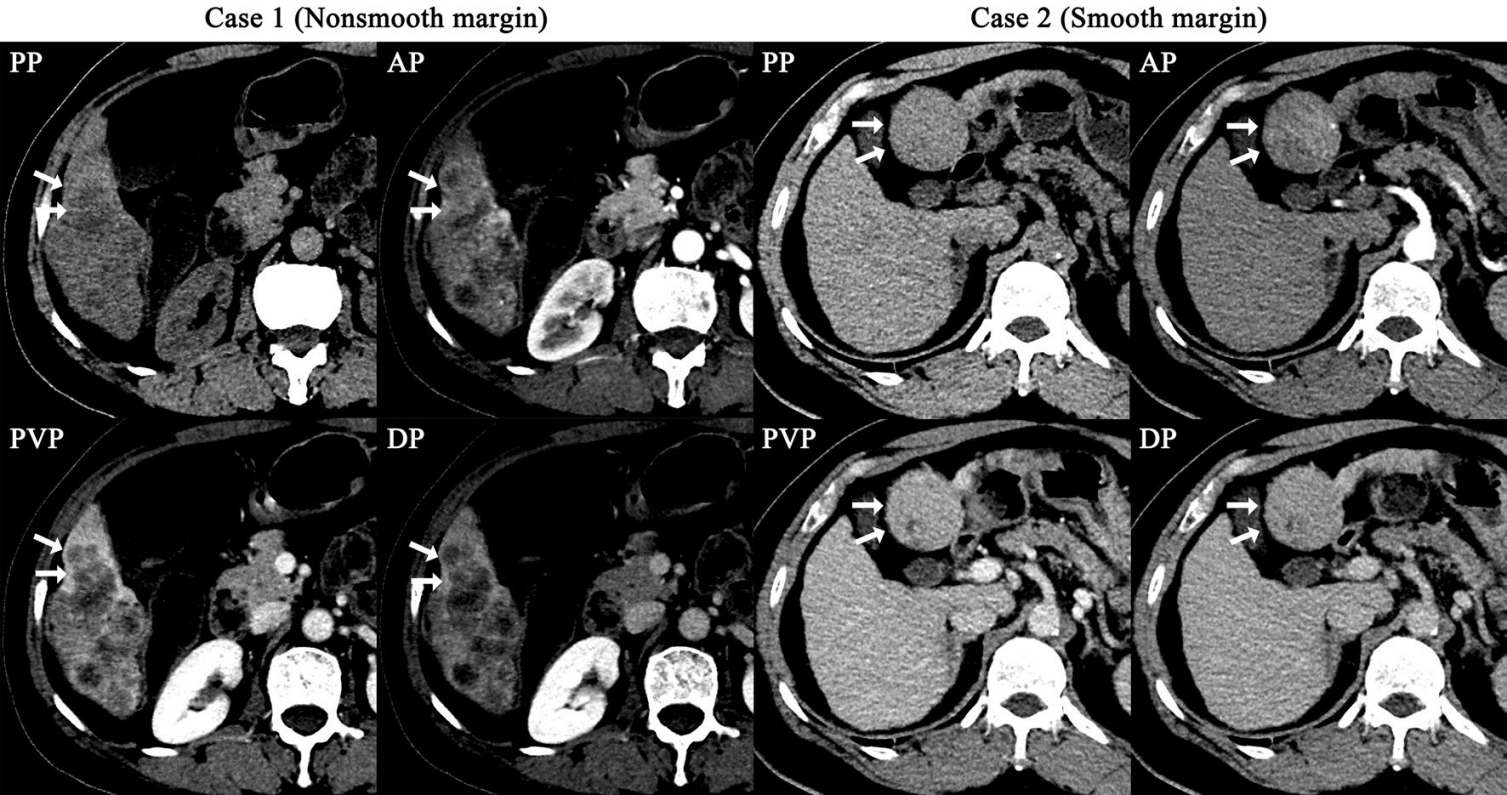
The most important feature (the margin of a tumour) for predicting MVI status in the RRC model (Case 1 with a nonsmooth tumour margin vs. Case 2 with a smooth tumour margin). Case 1 is MVI positive, and Case 2 is MVI negative

To identify the most vital features in the preoperative assessment of MVI status, all variables, including 17 radiological features, 7302 radiomics features and 19 baseline characteristics of patients, were evaluated for their importance in predicting MVI status by the XGBoost method. Finally, 129 features were found to contribute to the RRC model. Of all the variables, the tumour margin was ranked first and was the only radiological feature ranking in the top 15 features (Fig. 4), and *α*-fetoprotein (AFP) level was ranked 4th and was the only baseline characteristic ranking in the top 15 features. The remaining important variables were radiomics features (Table 4).

**Table 4:** The 15 most important features for MVI classification in the RRC Model

The 15 most important features in the RRC Model	Performance of the features in the model
TUMOR_MARGIN	0.079
Arterial_Phase_dilation-log-sigma-4-0-mm-3D_glcm_Imc1	0.037
Delay_Phase_dilation-wavelet-LHH_glszm_GrayLevelNonUniformity	0.037
Delay_Phase_dilation-wavelet-LHH_glszm_SizeZoneNonUniformity	0.026
AFP	0.026
Venous_Phase_dilation-wavelet-LLL_glcm_Imc2	0.021
Arterial_Phase-log-sigma-5-0-mm-3D_gldm_LowGrayLevelEmphasis	0.016
Venous_Phase-original_glcm_SumEntropy	0.016
Venous_Phase-wavelet-HHH_firstorder_Mean	0.016
Delay_Phase-wavelet-LLH_glszm_SmallAreaEmphasis	0.016
Delay_Phase_dilation-wavelet-LLH_firstorder_Skewness	0.016
Delay_Phase_dilation-wavelet-LLL_firstorder_InterquartileRange	0.016
Delay_Phase_dilation-wavelet-LLL_firstorder_Uniformity	0.016
Arterial_Phase-wavelet-LLL_glszm_ZonePercentage	0.011
Venous_Phase-log-sigma-2-0-mm-3D_gldm_SmallDependenceLowGrayLevelEmphasis	0.011

In the Radiological Model, the five most important radiological features are as follows: margin of tumor, internal arteries, hypo-dense halo, peritumoral enhancement and lobes involved.

### Comparison of the predictive models by 3D-CNN and XGBoost

In the training set, the 3D-CNN Model had the highest AUROC value among the other models, whereas the AUROC value of the Radiological Model was the lowest. The AUROC value of the Radiological Model was lower than that of the Radiomics Model (0.900 vs. 0.951, *p* = 0.0026). The AUROC value of the Radiomics Model was comparable to that of the RRC Model (0.951 vs. 0.965, *p* = 0.0523), which demonstrated that the radiological features and clinical variables did not provide significant added value to the Radiomics Model. The AUROC value of the 3D-CNN Model was higher than that of the Radiomics Model (0.980 vs. 0.951, *p* = 0.0148). However, there was no significant difference between the AUROC value of the 3D-CNN Model and that of the RRC Model (0.980 vs. 0.965, *p* = 0.1444).

In the validation set, there were no significant differences among the AUROC values of the several predictive models. The AUROC values of the Radiomics Model and the Radiological Model were 0.888 vs. 0.873, *p* = 0.73. The AUROC values of the Radiomics Model and the RRC Model were 0.888 vs. 0.897, *p* = 0.72. The AUROC values of the 3D-CNN Model and the Radiomics Model were 0.906 vs. 0.888, *p* = 0.66. The AUROC values of the 3D-CNN Model and the RRC Model were 0.906 vs. 0.897, *p* = 0.83.

### Recurrence-free survival analysis based on predicted MVI status

**Fig. 5:**
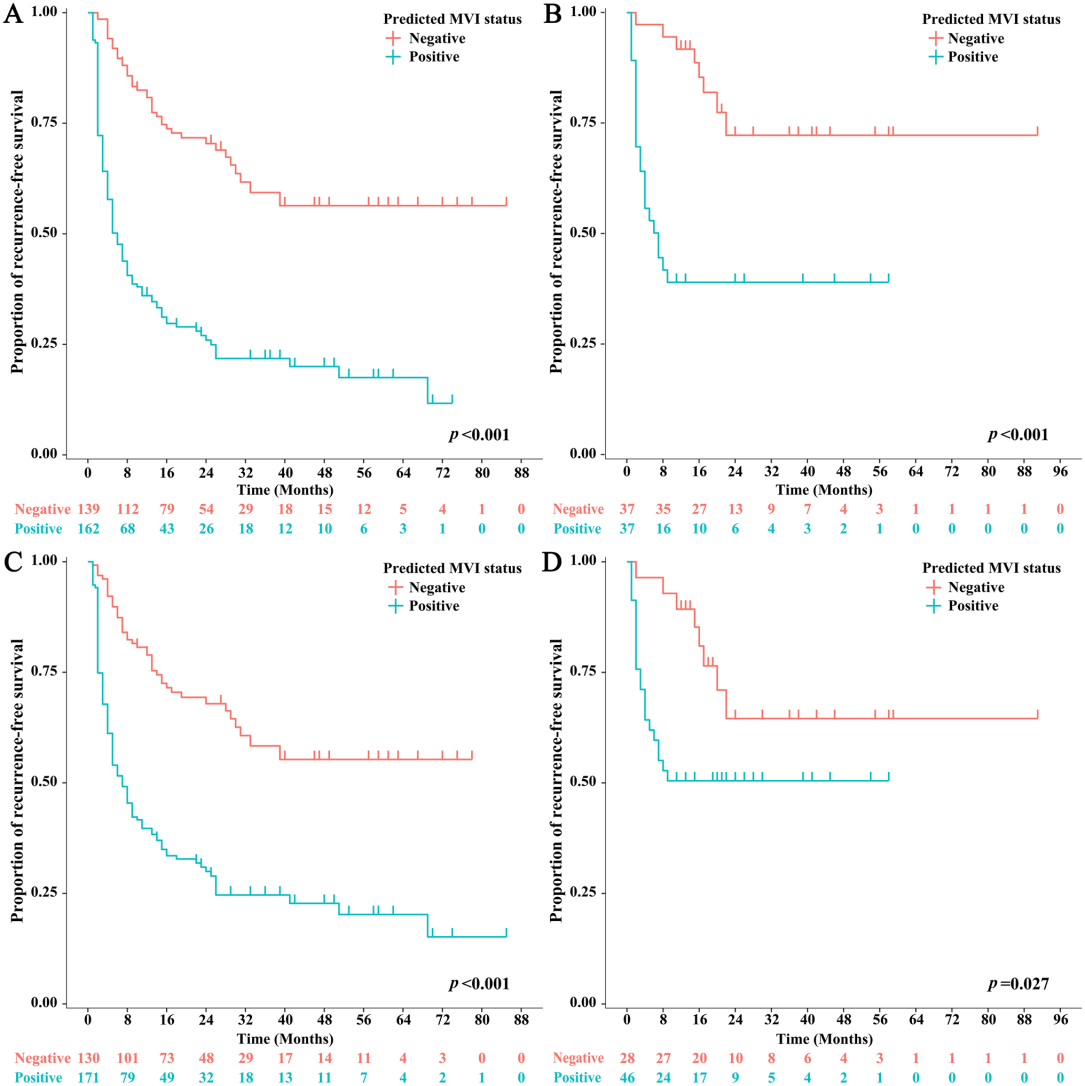
Recurrence-free survival analyses based on predicted MVI status by **a** the RRC Model in the training set; **b** the RRC Model in the validation set; **c** the 3D-CNN Model in the training set; and **d** the 3D-CNN Model in the validation set

The median recurrence-free survival (RFS) of the entire cohort was 22 months. The median RFS of patients with MVI was 6 months. The median RFS of patients without MVI was not available because less than half of the patients experienced recurrence. Kaplan–Meier survival analyses were performed based on the MVI status predicted by the RRC Model and 3D-CNN Model (Fig. 5) within the training set and the validation set. Based on the MVI status predicted by the RRC Model and the 3D-CNN Model, the mean RFS was significantly better in the predicted MVI-negative group than that in the predicted-MVI positive group (in the training set, RRC Model: 55.30 months vs. 19.99 months, *p* < 0.001; 3D-CNN Model: 50.24 months vs. 23.95 months, *p* < 0.001. In the validation set, RRC Model: 69.95 months vs. 24.80 months, *p* < 0.001; 3D-CNN Model: 64.06 months vs. 31.05 months, *p* = 0.027).

## Discussion

A preoperative noninvasive assessment of MVI may be essential to guide treatment strategies. In this study, we developed models based on image analysis by XGBoost and 3D-CNN, which may enhance the accuracy of preoperative non-invasive assessment of MVI in HCC patients. These machine learning models shown considerable efficacy in identifying MVI preoperatively.

Several studies have utilized radiological features or radiomics features to predict the status of MVI in HCC. Studies have reported that radiological features like the tumour margin, internal arteries, peritumoural enhancement and hypodense halos are essential in predicting MVI (Banerjee et al. 2015; Renzulli et al. 2016, 2018; Zheng et al. 2017), which is consistent with the current study. With the development of computer-assisted diagnosis methods, radiomics analysis has also been adopted to predict MVI status in HCC. In the study by Xu et al. (2019), they developed a regression model based on radiological features, clinical variables and radiomics features to predict MVI status and achieved an AUROC of 0.889 in the internal test set. In the current study, we also developed the RRC Model based on radiological features, radiomics features and clinical variables using a machine learning method, namely XGBoost. The RRC Model achieved an AUROC of 0.897 in the internal validation set, which is similar to Xu et al. study. We also developed models based on radiological features or radiomics features, and there were no significant differences between the Radiological Model and the Radiomics Model. We believe that each of the two methods has its own advantages. Radiological features are easy to understand and practical in clinical work, however, the accuracy of abstract of these features rely on experience of radiologists. Radiomics features are pre-defined by experts and quantified by computer, which are independent of experience of radiologists.

The most important highlight of the current study is that to the best of our knowledge, this is the first study to develop an MVI predictive model based on image analysis using machine learning methods (XGBoost and a convolutional neural network). Both of the models showed substantial efficacy in identifying MVI status. For the construction of the RRC Model developed by XGBoost, we collected comprehensive and detailed data including radiological features, radiomics features (based on manual segmentation) and clinical variables, which required extensive work and manpower. Radiomics is now an advanced technique used for image analysis. However, the shortcoming of radiomics analysis is that the method is based on hand-crafted feature extractors, which rely on expert definition and thus do not represent the most optimal option (Hosny et al. 2018). In contrast, the most important advantage of the 3D-CNN Model is that the model achieved high efficacy in identifying MVI status automatically with minimal manpower, time and materials. For the construction of the 3D-CNN Model, we needed only to input images, and clinical data, radiological features or radiomics features did not need to be collected. This significant efficacy accompanied by high efficiency is the primary driver to advance the application of artificial intelligence in medicine. Another innovative point of the current study is that we provided a new means to explain how deep learning can identify MVI. The greatest deficiency of deep learning or a CNN is the lack of interpretability. End-to-end predictive models are common in previous studies utilizing deep learning. To solve this problem, we extracted the output of the second last decision layer as the features to represent the CNN model. We evaluated the performance of the CNN model regarding the identification of some valuable features of HCC (radiological features and radiomics features) in the CT images, and the results were satisfactory, indicating that the CNN model can predict the status of MVI partly based on the explainable features utilized in daily clinical work.

Limitations existed in the current study. First, the accuracy of the 3D-CNN Model in identifying radiological features and radiomics features requires further improvement as we did not intend to construct a model dedicated to identifying these features. We believe that such models can be easily developed in the future, which may substantially reduce the workload of radiologists. Second, this is a single-centre study with a relatively small sample size, and the results therefore require further validation.

In conclusion, we proposed state-of-the-art models based on image analysis by XGBoost and deep learning to provide a preoperative noninvasive assessment method for MVI in HCC patients. The 3D-CNN model showed considerable efficacy in identifying MVI preoperatively with minimal manpower, time and material requirements. This model may facilitate decision making in HCC treatment. We believe that our model may have a substantial impact on the evaluation of tumour stages and the selection of appropriate treatments for HCC patients. Furthermore, this model may also advance the application of artificial intelligence in the area of hepatology. The validity of the model, as well as the long-term outcomes of patients who received treatments based on the model, requires further investigation.

## Electronic supplementary material

Below is the link to the electronic supplementary material.

Supplementary file1 (DOCX 22 kb)

Supplementary file2. Supplemental Figure 1. Flow chart (PDF 38 kb)

Supplementary file3. Supplemental Figure 2. Graphical abstract (TIF 9713 kb)

Supplementary file4 (DOCX 18 kb)

Supplementary file5 (DOCX 16 kb)

## Data Availability

The data and material are available from the corresponding author upon reasonable request.

## Abbreviations

AFP: *α*-Fetoprotein
AUROC: Area under receiver operating characteristic curve
AUPRC: Area under precision-recall curve
CI: Confidence interval
HCC: Hepatocellular carcinoma
HR: Hepatic resection
LT: Liver transplantation
MVI: Microvascular invasion
RFS: Recurrence-free survival
RRC: Radiomics-Radiological-Clinical
3D-CNN: Three-dimensional convolutional neural network
XGBoost: EXtreme Gradient Boosting
AP: Arterial phase
PVP: Portal-venous phase
DP: Delayed phase
VOI: Volume-of-interest

## Code availability

The custom code used to analyse the data is available from the corresponding author upon reasonable request.
