# Supplementary material for: Preoperative identification of microvascular invasion in hepatocellular carcinoma by XGBoost and deep learning
Source: J Cancer Res Clin Oncol. 2020 Aug 27;147(3):821–33. doi: 10.1007/s00432-020-03366-9 (PMC7873117; doi:10.1007/s00432-020-03366-9)
Supplement: Supplementary file 1 — Supplementary file1 (DOCX 22 kb) [file 432_2020_3366_MOESM1_ESM.docx]

**Supplemental Methods**

***Pathological assessment of MVI***

A pathological biopsy was collected at the positions of the nontumoural part of the tumour margin. Paracancerous liver tissues (at a distance of ≤ 1 cm and > 1 cm from the edge of the cancer) were also obtained. Finally, at least 12 sections of nontumoural hepatic parenchyma were examined by two experienced pathologists. MVI was defined as tumour tissue within a vascular space lined by endothelium that was visible only by microscopy(1).

***CT imaging protocol***

A four-phase DCE CT imaging protocol including the precontrast phase (PP), arterial phase (AP), portal venous phase (PVP), and delayed phase (DP) was performed using a 64- or 320-detector CT scanner (Activion 16, Aquilion or Aquilion ONE; Toshiba Medical Systems, Ottawara, Japan), and the following acquisition parameters were used: tube rotation time, 0.5 seconds; pitch factor, 0.828; field of view, 35 cm×35 cm; matrix 512×512; tube voltage, 120 kV (peak) and automatic tube current modulation.

The three phases were contrast material-enhanced phases with the same scanning range after the intravenous administration of 1 ml/kg of low osmolar nonionic contrast medium (iodine concentration 300 mg I/ml) (Iopromide, Schering, Berlin, Germany) in the right antecubital vein at a rate of 3 mL/sec, followed by a 10-mL saline chaser.

A bolus tracking technique was performed to acquire hepatic AP images. The AP was scanned 15 seconds after CT attenuation of the aorta at the level of the diaphragm had reached 200 HU. For the PVP, images were acquired 55-60 seconds after the contrast injection. The DP was scanned 3 mins after contrast injection. Each phase was reconstructed with a setting of 5-mm slice thickness.

***Predictive models based on XGBoost***

*Definitions of radiological feature*

The definitions of some radiological features are as follows. The internal arteries were defined as either the visible internal vessels that were irregular in contour and branched in a distorted fashion unlike normal progressive anatomic arborization or randomly distributed hyperattenuating and hypoattenuating regions in the AP.(2) The tumor margin was categorized as a smooth margin if it presented a smooth contour or a non-smooth margin if it presented as an irregular margin that had a budding portion at the periphery on the axial, coronary and sagittal images.(3) A hypodense halo was defined as a rim of hypoattenuation partially or completely circumscribing the tumor.(4) Peritumoral enhancement was defined as the existence of a detectable portion enhanced in the arterial phase, adjacent to the tumor border, later becoming isoattenuating on CT images as compared with the background liver parenchyma in the equilibrium phase.(3) An enhanced “capsule” was defined as a peripheral rim of smooth hyperenhancement in the portal venous or delayed phase.(3)

*Feature analysis and predictive model based on XGBoost*

We used the open source XGBoost package for MVI classification and feature importance analysis. Gradient tree boosting methods employ an ensemble of multiple decision trees to strengthen the classification power. Each decision tree is grown by selecting the most discriminative features from the large feature candidate pool. This process relieves tree-based methods from the onerous feature selection process and allows the classifier to directly interact with the features. The feature selection process of the algorithm facilities the analysis of the features that most impact the classifier and thus provides us with a method of investigating the pathogenic mechanism hidden within the images.

***Deep learning: the 3D-CNN Predictive Model***

*Details about 3D-CNN Model*

Our model takes three 16 x 64 x 64 patches (sampled from the central tumor areas of three-phase scans with 10% randomness) as input, and passes it through several intermediate layers to extract deep features, which are further fused and fed to the decision layers to generate the final MVI assessment. We experimented with various choices of the network, including the convolutional kernel sizes, network depth, down sampling rates, etc, and the network architecture achieving the best result is summarized as Figure 1. This architecture learns deep semantics of the tumor regions, but maintaining lightweight yet efficient to alleviate the overfitting problem caused by small datasets in the medical image domain.

We performed deep learning by using a computer with 4 NVIDIA TESLA P40 graphicses processing units, 56 Intel(R) Xeon(R) CPU E5-2680 v4 @ 2.40GHz central processing units, and 503 GB of random access memory. The Python programming language and PyTorch 1.1.0 (https://pytorch.org/) framework were used to run our designed neural networks. We use the Adam optimizer and train our model for 80 epochs. The batch size is 64 (16 per GPU) with 4 NVIDIA Tesla P40 GPUs. The learning rate is set to 0.0001 with a weight decay of 0.0005.

*Interpretability of the 3D-CNN Model*

One of the major criticisms of the deep CNN approach is the lack of interpretability, meaning that the system struggles to provide evidence to support the given decision. To mitigate this problem, methods such as gradient-weighted class activation mapping (Grad-CAM) were proposed to provide class-discriminative heatmaps as visual explanations of the CNN model predictions.[5] However, such primitive visual indications were difficult to summarize or link with the high-level knowledge of radiologists. In this study, we proposed an explanation method by exploiting the correlation between the CNN features and other interpretable features, e.g., routine preoperative laboratory examination values, radiological features, and radiomics features. We extracted the output of the second last decision layer as the features to represent the CNN model. We selected the top 15 important variables selected by the XGBoost method and organized them as the targets. To measure their correlation, an intuitive idea was to use the extracted CNN features (which are good at predicting MVI) to predict these interpretable features. A high prediction accuracy means that the established CNN model has encoded the interpretable characteristics to assist in the decision-making process.

**REFERENCES**

1. Roayaie S, Blume IN, Thung SN, Guido M, Fiel MI, Hiotis S, Labow DM, Llovet JM, Schwartz ME. A system of classifying microvascular invasion to predict outcome after resection in patients with hepatocellular carcinoma. Gastroenterology 2009;137(3):850-855. doi: 10.1053/j.gastro.2009.06.003

2. Nino-Murcia M, Olcott EW, Jeffrey RB, Jr., Lamm RL, Beaulieu CF, Jain KA. Focal liver lesions: pattern-based classification scheme for enhancement at arterial phase CT. Radiology 2000;215(3):746-751. doi: 10.1148/radiology.215.3.r00jn03746

3. Lee S, Kim SH, Lee JE, Sinn DH, Park CK. Preoperative gadoxetic acid-enhanced MRI for predicting microvascular invasion in patients with single hepatocellular carcinoma. J Hepatol 2017;67(3):526-534. doi: 10.1016/j.jhep.2017.04.024

4. Segal E, Sirlin CB, Ooi C, Adler AS, Gollub J, Chen X, Chan BK, Matcuk GR, Barry CT, Chang HY, Kuo MD. Decoding global gene expression programs in liver cancer by noninvasive imaging. Nat Biotechnol 2007;25(6):675-680. doi: 10.1038/nbt1306

5. Selvaraju RR, Cogswell M, Das A, Vedantam R, Parikh D, Batra D. Grad-cam: Visual explanations from deep networks via gradient-based localization. Proceedings of the IEEE International Conference on Computer Vision2017; p. 618-626.
