# Supplementary figures and images for: Preoperative identification of microvascular invasion in hepatocellular carcinoma by XGBoost and deep learning
Source: J Cancer Res Clin Oncol. 2020 Aug 27;147(3):821–33. doi: 10.1007/s00432-020-03366-9 (PMC7873117; doi:10.1007/s00432-020-03366-9)

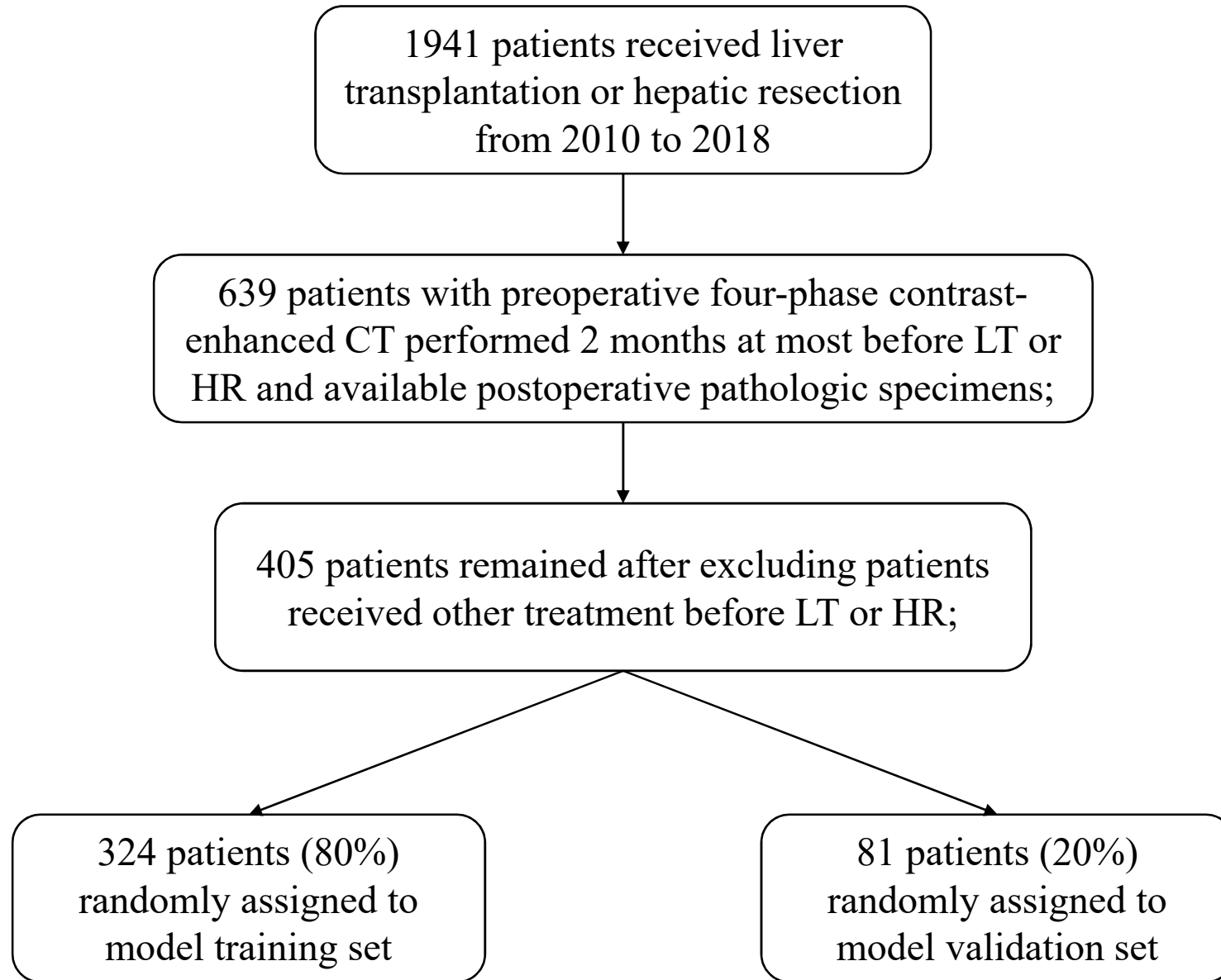

Supplement: Supplementary file 2 — Supplementary file2. Supplemental Figure 1. Flow chart (PDF 38 kb) [file 432_2020_3366_MOESM2_ESM.pdf]

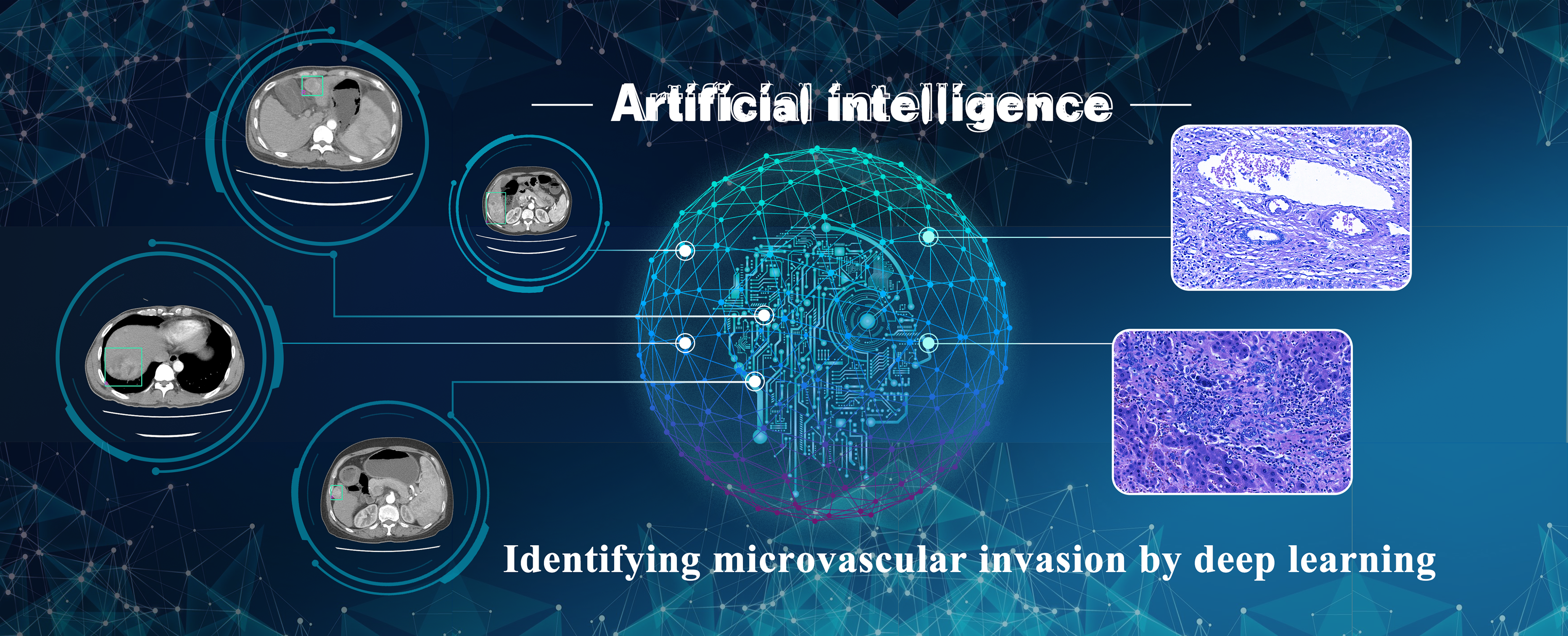

Supplement: Supplementary file 3 — Supplementary file3. Supplemental Figure 2. Graphical abstract (TIF 9713 kb) [file 432_2020_3366_MOESM3_ESM.tif]
