# Supplementary material for: Preoperative identification of microvascular invasion in hepatocellular carcinoma by XGBoost and deep learning
Source: J Cancer Res Clin Oncol. 2020 Aug 27;147(3):821–33. doi: 10.1007/s00432-020-03366-9 (PMC7873117; doi:10.1007/s00432-020-03366-9)
Supplement: Supplementary file 4 — Supplementary file4 (DOCX 18 kb) [file 432_2020_3366_MOESM4_ESM.docx]

**Supplementary Table 1: Clinical Variables Stratified by MVI Status in the Training Set and Validation Set**

|  | Training set | | | Validation set | | |  |
| --- | --- | --- | --- | --- | --- | --- | --- |
| Variable | MVI negative (n=148) | MVI positive (n=176) | *p* | MVI negative (n=37) | MVI positive (n=44) | *p* | *p*^a^ |
| Age | 49.6±14.4 | 47.2±12.7 | 0.13 | 47.4±13.4 | 47.8±11.5 | 0.88 | 0.56 |
| Sex (Male) | 117 | 158 | 0.007 | 33 | 36 | 0.35 | 0.95 |
| Diabetes (Yes) | 15 | 14 | 0.49 | 3 | 4 | 0.88 | 0.93 |
| Background disease of liver |  |  | 0.27 |  |  | 0.34 | 0.94 |
| HBV infection | 130 | 147 |  | 30 | 39 |  |  |
| Other | 18 | 29 |  | 7 | 5 |  |  |
| Surgery Type |  |  | 0.67 |  |  | 0.93 | 0.36 |
| HR | 127 | 148 |  | 33 | 39 |  |  |
| LT | 21 | 28 |  | 4 | 5 |  |  |
| AFP |  |  | <0.001 |  |  | 0.004 | 0.45 |
| <10 | 66 | 32 |  | 16 | 7 |  |  |
| 10-100 | 34 | 33 |  | 4 | 7 |  |  |
| 100-400 | 14 | 31 |  | 6 | 10 |  |  |
| 400-1000 | 12 | 6 |  | 4 | 2 |  |  |
| >1000 | 22 | 74 |  | 7 | 18 |  |  |
| ALT | 44.3±43.7 | 53.0±62.9 | 0.06 | 48.1±52.4 | 97.2±253.7 | 0.25 | 0.38 |
| AST | 44.5±44.9 | 64.4±81.9 | 0.02 | 41.6±29.5 | 136.2±434.3 | 0.19 | 0.41 |
| PLT | 172.7±87.4 | 189.7±80.9 | 0.16 | 160.1±84.5 | 189.5±95.4 | 0.15 | 0.12 |
| PT | 14.6±2.7 | 14.2±1.7 | 0.05 | 14.6±2.8 | 14.0±1.8 | 0.23 | 0.15 |
| INR | 1.15±0.29 | 1.10±0.17 | 0.06 | 1.16±0.29 | 1.10±0.18 | 0.25 | 0.24 |
| FBG | 3.10±1.27 | 3.53±1.25 | 0.004 | 2.92±1.23 | 3.42±1.27 | 0.08 | 0.98 |
| ALB | 39.6±4.9 | 38.7±4.8 | 0.20 | 39.2±5.6 | 39.2±5.6 | 0.97 | 0.11 |
| TBIL | 22.8±37.4 | 32.3±128.4 | 0.39 | 23.4±24.9 | 29.2±71.3 | 0.64 | 0.97 |
| SCr | 56.4±37.0 | 61.9±34.1 | 0.37 | 97.6±187.9 | 53.7±35.3 | 0.13 | 0.61 |
| Child-Pugh class |  |  | 0.06 |  |  | 0.09 | 0.10 |
| A | 128 | 147 |  | 28 | 37 |  |  |
| B | 15 | 28 |  | 8 | 3 |  |  |
| C | 5 | 1 |  | 1 | 4 |  |  |
| MELD score | 8.3±4.1 | 7.7±4.1 | 0.11 | 8.3±5.4 | 7.3±4.8 | 0.35 | 0.38 |
| *p*^a^, *p* value for the test between the training set and the validation set.  HBV (Hepatitis B Virus), HR (hepatic resection), LT (liver transplantation), AFP (α-fetoprotein), ALT (alanine aminotransferase), AST (aspartate aminotransferase), PLT (platelet), PT (prothrombin time), INR (international normalized ratio), FBG (fibrinogen), ALB (albumin), TBIL (total bilirubin), SCr (serum creatinine), MELD (Model for end-stage liver disease) | | | | | | | |
