# Supplementary material for: Preoperative identification of microvascular invasion in hepatocellular carcinoma by XGBoost and deep learning
Source: J Cancer Res Clin Oncol. 2020 Aug 27;147(3):821–33. doi: 10.1007/s00432-020-03366-9 (PMC7873117; doi:10.1007/s00432-020-03366-9)
Supplement: Supplementary file 5 — Supplementary file5 (DOCX 16 kb) [file 432_2020_3366_MOESM5_ESM.docx]

**Supplementary Table 2: Interpretability of the 3D-CNN Model: Performance of the 3D-CNN Model in Predicting Interpretable Features for MVI Classification**

| The 15 most important features in the RRC Model | AUROC (Training set) | AUROC (Validation set) | Specificity | Sensitivity |
| --- | --- | --- | --- | --- |
| TUMOR_MARGIN | 0.867 | 0.789 | 0.846 | 0.655 |
| Arterial_Phase_dilation-log-sigma-4-0-mm-3D_glcm_Imc1 | 0.827 | 0.824 | 0.818 | 0.811 |
| Delay_Phase_dilation-wavelet-LHH_glszm_GrayLevelNonUniformity | 0.856 | 0.916 | 0.906 | 0.821 |
| Delay_Phase_dilation-wavelet-LHH_glszm_SizeZoneNonUniformity | 0.781 | 0.887 | 0.772 | 0.875 |
| AFP | 1 | 0.614 | 0.622 | 0.614 |
| Venous_Phase_dilation-wavelet-LLL_glcm_Imc2 | 0.719 | 0.708 | 0.762 | 0.641 |
| Arterial_Phase-log-sigma-5-0-mm-3D_gldm_LowGrayLevelEmphasis | 0.8 | 0.823 | 0.723 | 0.824 |
| Venous_Phase-original_glcm_SumEntropy | 0.79 | 0.842 | 0.8 | 0.774 |
| Venous_Phase-wavelet-HHH_firstorder_Mean | 0.85 | 0.956 | 0.887 | 0.929 |
| Delay_Phase-wavelet-LLH_glszm_SmallAreaEmphasis | 0.844 | 0.806 | 0.63 | 0.914 |
| Delay_Phase_dilation-wavelet-LLH_firstorder_Skewness | 0.921 | 0.919 | 0.933 | 0.806 |
| Delay_Phase_dilation-wavelet-LLL_firstorder_InterquartileRange | 1 | 0.569 | 0.76 | 0.419 |
| Delay_Phase_dilation-wavelet-LLL_firstorder_Uniformity | 0.819 | 0.812 | 0.833 | 0.689 |
| Arterial_Phase-wavelet-LLL_glszm_ZonePercentage | 0.873 | 0.777 | 0.694 | 0.8 |
